# TOGGLE: toolbox for generic NGS analyses

**DOI:** 10.1186/s12859-015-0795-6

**Published:** 2015-11-09

**Authors:** Cécile Monat, Christine Tranchant-Dubreuil, Ayité Kougbeadjo, Cédric Farcy, Enrique Ortega-Abboud, Souhila Amanzougarene, Sébastien Ravel, Mawussé Agbessi, Julie Orjuela-Bouniol, Maryline Summo, François Sabot

**Affiliations:** UMR DIADE IRD/UM, 911 Avenue Agropolis, Montpellier Cedex 5, F-34934 France; UMR AGAP CIRAD/INRA/SupAgro, TA A-108/03 - Avenue Agropolis, Montpellier Cedex 5, F-34398 France; UMR-BGPI CIRAD TA A-54/K, Campus International de Baillarguet, Montpellier Cedex 5, F-34398 France; ADNid, Cap Alpha, Avenue de l’Europe, Clapiers, F-34830 France

**Keywords:** NGS, Toolbox, Pipeline, Flexible

## Abstract

**Background:**

The explosion of NGS (Next Generation Sequencing) sequence data requires a huge effort in Bioinformatics methods and analyses. The creation of dedicated, robust and reliable pipelines able to handle dozens of samples from raw *FASTQ* data to relevant biological data is a time-consuming task in all projects relying on NGS. To address this, we created a generic and modular toolbox for developing such pipelines.

**Results:**

TOGGLE (*TOolbox for Generic nGs anaLysEs*) is a suite of tools able to design pipelines that manage large sets of NGS softwares and utilities. Moreover, TOGGLE offers an easy way to manipulate the various options of the different softwares through the pipelines in using a single basic configuration file, which can be changed for each assay without having to change the code itself. We also describe one implementation of TOGGLE in a complete analysis pipeline designed for SNP discovery for large sets of genomic data, ready to use in different environments (from a single machine to HPC clusters).

**Conclusion:**

TOGGLE speeds up the creation of robust pipelines with reliable log tracking and data flow, for a large range of analyses. Moreover, it enables Biologists to concentrate on the biological relevance of results, and change the experimental conditions easily. The whole code and test data are available at https://github.com/SouthGreenPlatform/TOGGLE.

**Electronic supplementary material:**

The online version of this article (doi:10.1186/s12859-015-0795-6) contains supplementary material, which is available to authorized users.

## Background

Since the appearance of Next Generation Sequencing (NGS) technologies, a large number of bioinformatics softwares and methods have been developed and publicly released to work with these massive sequence data [1, 2]. Depending on the type of data and experiments, multiple steps may be required such as quality control, adapter removal or trimming or both, mapping, variant detection, read counting and so on, and dedicated software may be used for each step. Thus, biologists have to perform a lot of chained manipulations in order to obtain the final information for their research. The vast majority of bioinformatics softwares are command-lines tools, generally designed to work on powerful computers and High-Performance Calculation (HPC) infrastructures. Bioinformaticians have developed dedicated pipelines for specific experiments, using those bioinformatics softwares, as well as combinations of home-made filters (using conditional *if*) and loops (using the classical *while*) [3, 4]. However, those scripts are generally monolithic, in the sense that they cannot easily be transferred from one installation to another, or from one experimental subject to the following (different species, different conditions, etc.). Moreover, even if some flexibility is offered to the user by modification of several options before launching, these modifications are generally not trivial, requiring either a long list in the command line, or provision of a complex option file. In addition, writing a new pipeline *from scratch* is labor-intensive and time-consuming, especially if all safeguards for file format, software execution, or file transfer are implemented each time.

The TOGGLE suite (for *TOolbox for Generic nGs anaLysEs*) was developed to optimize the creation of new NGS analysis workflows. What we propose here is a set of packages designed for fast implementation of robust and reliable pipelines. Each package represents either a NGS software manipulation or a set of dedicated tools (see Table 1). These packages are written in *Perl*, with unitary modules the most generic possible. TOGGLE is able to manage hundreds of samples at once, and is not limited to one application. It can be used on DNAseq, RNA-Seq, smallRNA, GBS, and any other sequence data. It can be installed on a variety of infrastructure, from a simple laptop computer to a large HPC cluster. Finally, the TOGGLE assays and options are based on a unique configuration file, that can be easily changed and adapted to each run. The tool and test data are available on GitHub: https://github.com/SouthGreenPlatform/TOGGLE. A *Docker* image for immediate installation is available (http://bioinfo-web.mpl.ird.fr/toggle/toggle.tgz), as well as an user-space install script (http://bioinfo-web.mpl.ird.fr/toggle/installTOGGLE.sh).

**Table 1:** List of the different packages and main modules. If any other modules exist but are not presented here, multiple dots are shown

Packages (nb of modules)	Modules	Functions
bwa (5)	bwaIndex	Indexing the reference Fasta file
	bwaAln	Create the alignment for *FASTQ* sequences
	bwaSampe	Create the *SAM* file for pair-end sequences
	bwaSamse	Create the *SAM* file for single-end sequences
	...	...
cutadapt (2)	CreateConfFile	Create the specific *cutadapt* configuration file
	execution	Execute *cutadapt* command
fastqc (6)	execution	Run *FastQC* software
	parse	Parse the *FastQC* results
	...	...
gatk (8)	gatkVariantFiltrator	Filter the different SNP based on specific options
	gatkHaplotypeCaller	Call the haplotype of each individual based on a *BAM* file
	...	...
pairing (4)	pairRecognition	Allow the recognition of pairs in a set of *FASTQ* files
	repairing	Reorganize in pair two *FASTQ* files, extract single sequences
picardTools (3)	picardToolsMarkDuplicates	Mark/eliminate different types of duplicate in a *BAM* file
	picardToolsCreateSequenceDictionnary	Create the “.dict” file of the reference
	picardToolsSortSam	Sort the *SAM* file
	...	...
samTools (10)	samToolsSort	Sort the *SAM*/*BAM* file
	samToolsIndex	Index the *SAM*/*BAM* file
	mergeHeader	Merge the header of multiples *SAM*/*BAM* into a single one
	...	...
toolbox (73)	exportLog	Export information into log files
	checkFile	Check if a file exists, is readable/writable, and is not empty
	existsDir	Check if a directory exists
	makeDir	Create a new directory
	readDir	Read the content of a directory
	extractPath	Extract the complete path of a file
	extractName	Create a readgroup from a file name
	readFileConf	Read the configuration file and return a hash
	extractOptions	Provide the options for a given software from the hash
	run	Run the command line given in argument
	checkSamOrBamFormat	Check if a file is a true *SAM*/*BAM* format
	extractHashSoft	Extract specific options for a given tool
	checkNumberByWC	Provide the number of sequence in a given *FASTQ* file
	checkEncodeByASCIIcontrol	Check the format of encoding in a *FASTQ* file
	changeEncode	Provide a wrapper to change the encoding of a *FASTQ* file
	...	...

## Implementation/results and discussion

### Packages and modules

We designed TOGGLE in a suite of more than 10 packages, gathering more than 60 different modules (functions or calling), which can be used independantly to quickly implement pipelines for NGS analyses. Each package represents either the use of a software suite (such as *bwa* [5] or *Genome Analysis ToolKit GATK* [6]), or a set of tools dedicated to a given operation or more general tools. Modules are *Perl* code wrappers for a central command or function, with a validation system for the entry file format, running command, and output (if any). Each module starts generally by calling the file format validation modules from the *toolbox.pm* package (for *FASTQ*, *SAM* and others), and then by sending a specific command line to the *toolbox::run* module. This module launches the command through the UNIX system, and will return either a given value for correct execution, a warning, or an error.

**Fig. 1:**
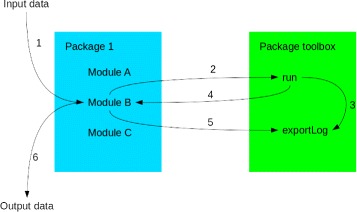
Software execution presentation. (1) Input data are submitted to a given Module. (2) The Module will construct a command line to *toolbox::run*. (3) The text of the command line and the run report are sent to *toolbox::exportLog*. (4) The output of the command (ok, error or warning) is sent back to the original Module. (5) The Module will send a report to *toolbox::exportLog*. (6) The output data are delivered from the Module

**Fig. 2:**
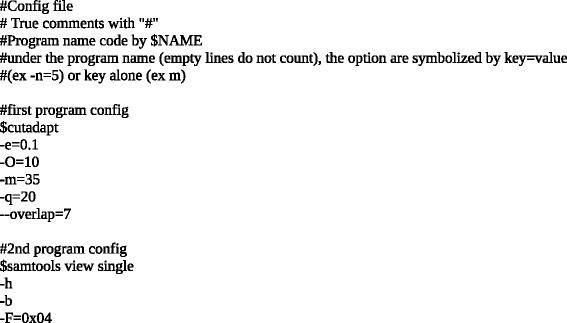
Softwares configuration file. The lines starting with “$” correspond to the name of the current module called (e.g. *bwa aln*). The lines just after list the option(s) associated with this call; the list of options is finished with an empty line. Lines starting with “ *♯*” are reserved for comments

The *toolbox.pm* package gathers general tools and functionalities. This package will, among other things, read the *software.config.txt* file, manage printing of log files printing (see below) and the system command for all modules (Fig. 1). The *software.config.txt* file can be used to manage the options for the different softwares and modules of TOGGLE. This file is constructed in a basic layout, as shown in Fig. 2, using the option representation specific for each software. If no specific parameters for a given software or module are provided in this file, the default values of the software/module will be used. The *software.config.txt* file (see Fig. 2) was developed with Biologist users in mind: this file is the only one to change for any option of any step of a given pipeline. The *localConfig.pm* package will inform of the different softwares paths (for *bwa* e.g.) on the user’s local machine or server. Users can adapt the paths in this package, and can also test different versions or installations of the same software without having to change the whole pipeline code. Only those three packages/files are mandatory when creating a new script using TOGGLE.

**Fig. 3:**
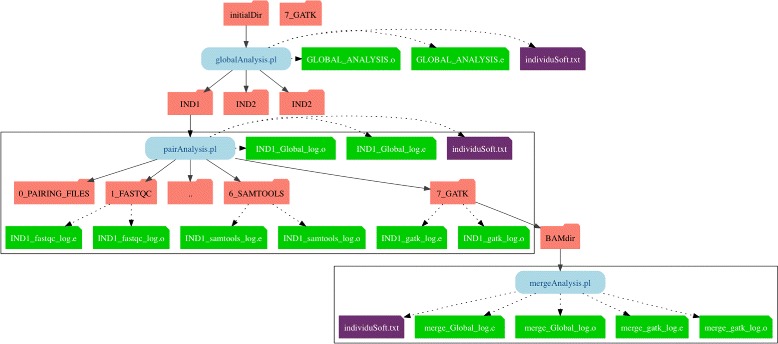
Directories tree structure. Representation of the tree of directories with the log files during the execution of *globalAnalysis.pl* pipeline, with the example of three individuals representing each possibility. The first one is a paired-end data, the second one is a single-end data, and the last one is a paired-end data which generate single reads during the cleaning step

A large part of the coding effort was to produce a logging system that can be scaled for hundreds of individual analyses run in parallel (Fig. 3). The dedicated *toolbox::exportLog* module will recover the value transmitted by other modules to generate log information: (*i*) the module call succeeded; (*ii*) the module call started correctly but finished incorrectly; (*iii*) the module was not able to execute the command. The last two types of information will be recovered in the error log file (“.e”). Correct execution of the command will be written to the output log file (“.o”). The log system allows the user to check step by step the progess of the analysis.

Adding new modules or packages is relatively simple, with a sample package provided with the complete suite. Linking to the different tools (running and logging system), as well as the replacement of a given module by another in a pipeline is easy to perform. A template for a new pipeline design is also provided.

### Example of implementation

**Fig. 4:**
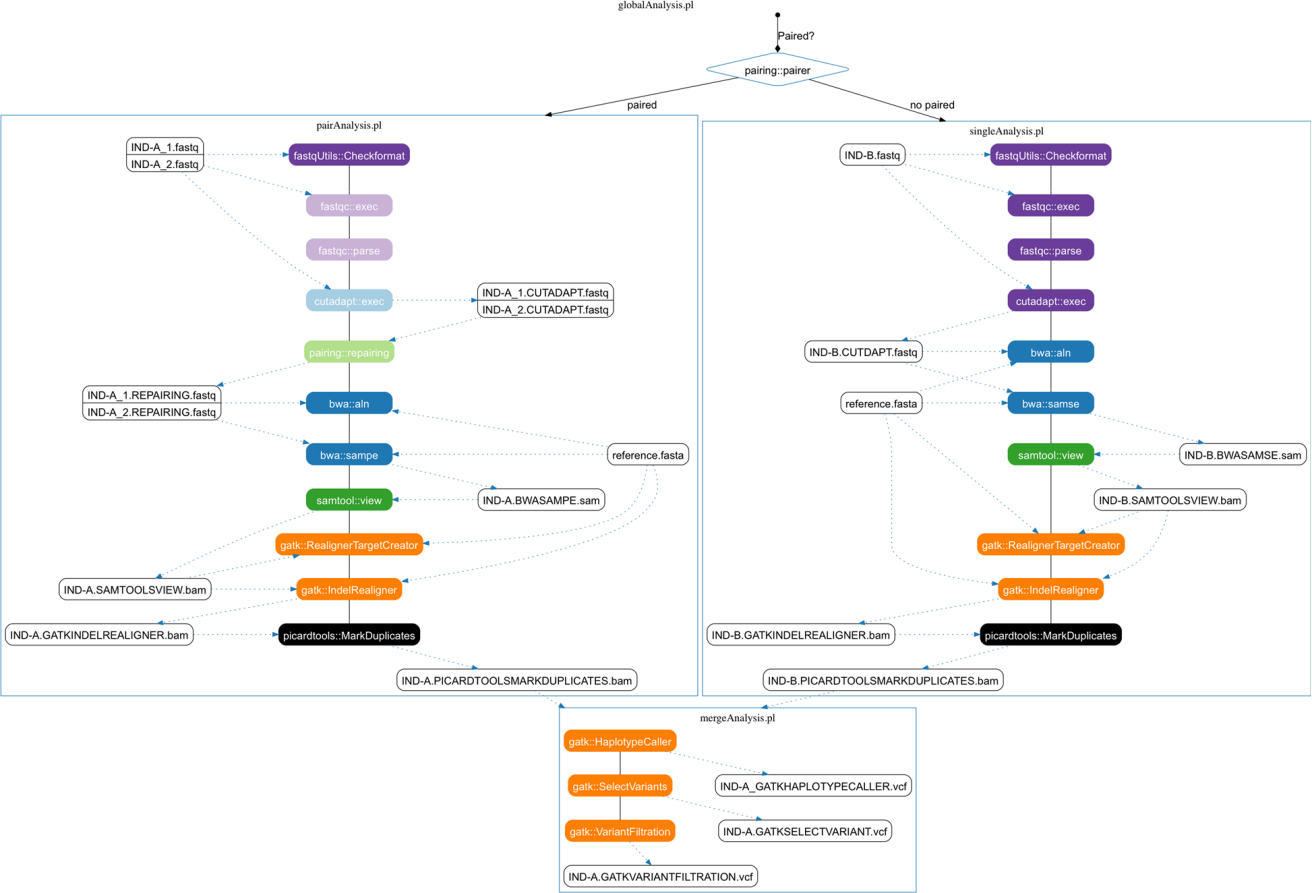
Pipeline DNAseq presentation. Basic overview of the *pairAnalysis.pl*, *singleAnalysis.pl*, *mergeAnalysis.pl* pipelines, and of the wrapping *globalAnalysis.pl* pipeline. Each colored box represents a given module, and each color a specific package. See text for the corresponding steps. A more complete figure is available on the TOGGLE website

All the modules can be arranged to easily generate robust pipelines for NGS analyses. As an example, we provide two functional pipelines: an RNAseq data one (not detailed here) and a DNAseq pipeline for single and paired-end *Illumina* genomic data for SNP and InDel detection in any eukaryotic diploid species. This latter, that we describe here in details, is based on four scripts: *pairAnalysis.pl*, *singleAnalysis.pl*, *mergeAnalysis.pl* and *globalAnalysis.pl* (Fig. 4). The first three can be launched independently, while the last, *globalAnalysis.pl*, is a turn-key pipeline which launches and manages the three others (see Additional file 1).

Both start with *FASTQ* format validation (purple boxes in Fig. 4), followed by a quality control using the *FastQC* software (yellow boxes in Fig. 4) [7], and a cleaning step performed by the *CutAdapt* software [8] (sand boxes). For *pairAnalysis.pl*, an additional “repairing” stage (red boxes in Fig. 4) re-associates the paired-end reads and exports the single-end reads (which may be produced by the cleaning step) to the *singleAnalysis.pl* script independently from *CutAdapt*. This step may be excluded when using the last version of *CutAdapt* and its pair-end mode, but this is not yet implemented in TOGGLE. After cleaning, both scripts follow the same stages again, with specific parameters: mapping using *bwa aln* (blue boxes; *bwa MEM* module is also available) [5]; sorting, cleaning and indexing of the *BAM* files using *PicardTools* [9] and *SAMtools* [10] (brown and green boxes, respectively); local realignment with the *GATK* (orange boxes) [6]; and finally removal of duplicates using *PicardTools* (brown boxes). The mapping is performed here using the *bwa aln/sampe/samse* back-track version. It is possible to switch to the *bwa mem* version by changing the code, as this module is already provided in the *bwa.pm* package. However, we decided to remain on the back-track version in this pipeline, as it is used in our labs and allows comparison with older mapping data. The data are then treated through the third script *mergeAnalysis.pl*, for Single Nucleotide Polymorphism (SNP) and Insertion/Deletions (InDels) calling using *HaplotypeCaller*, followed by the sorting and quality filtering of the obtained variants. All of these steps are performed using *GATK*, and are multiple sample variant calling. The final Variant Call Format (*VCF*) file is cleaned and filtered as much as possible through generic Bioinformatics tools, but subsequent specific analyses may be required to optimize it for specific purposes (e.g. SNP identification for CHiP creation, population genetics or breeding).

In order to automate these steps for multiple samples, we created the *globalAnalysis.pl* script (in currently two versions: one linear and the second one parallelized for *SGE* type architecture). This script will also organize the data into different directories with a specific tree structure (Fig. 3) defined to facilitate access to intermediate files.

The *globalAnalysis.pl* script will deal with a unique directory where the whole raw *FASTQ* data are stored, without correspondance of names between the two paired files (the forward can be named *Ulysse* and the reverse *Enterprise*). The script will use the *pairing::pairRecognition.pm* module (red boxes in Fig. 4) to associate files of paired-end data. The first sequence ID (specific for each run) is used to create the pairs, and we validated this association step of more than 150 paired and single files. It creates independent folders for each sample/individual, on which *pairAnalysis.pl* or *singleAnalysis.pl* will be launched according to the data type. After these analyses, *globalAnalysis.pl* will recover the final *BAM* file and its index for each sample, and will execute the *mergeAnalysis.pl* script as described earlier (see Fig. 4).

All those scripts are associated with an indicator file, *individuSoft.txt*, which inform which step of the analysis has been launched. This indicator file also permits to create the various logs files. Indeed we constructed different level of log files: at global level (GLOBAL_ANALYSIS_date on Fig. 3); at sample/individuals level (indX_global_log on Fig. 3), and at package per individual level (indX_package_log on Fig. 3).

This pipeline is a classical one for DNAseq analyses from *FASTQ* sequences to *BAM* files then *VCF* files, but with a lot of control regarding file structure and format, and is easy to manage in terms of specific and global options through the *software.config.txt* file. Moreover, it can be used with any reference genome (provided as a *FASTA* file). A RNAseq pipeline is also provided in the TOGGLE suite, based on a *TopHat* and *HTSeq* [11] approach (see Additional file 1).

### Parallelization

TOGGLE can manage parallelized jobs, and may be implemented on HPC cluster machines. Concurrent jobs can be launched using the *globalAnalysisSGE.pl* script for example, which will launch all individual *pairAnalysis.pl* (or *singleAnalysis.pl*) instances in different jobs. The specific SGE parameters for all individual jobs can be specified in the option file. For the moment, the main level of the paralellization is based on the sample level (each individual) only. We are conscious this is a main drawback compared to system such as *HugeSeq* [4] or *Churchill* [3] (see below). However, TOGGLE was designed to requested a very limited number of mandatory softwares/configuration outside of bioinformatics one. Thus it could be deployed on any kind of infrastructure. Nevertheless, in future versions we plan to develop a finer approach using a *MapReduce*/embarrassingly parallel approach, splitting each sample in multiples subsamples at the different levels (*FASTQ*, *SAM/BAM*, *VCF*), in a similar way to *Churchill* [3].

Still, the current scripts do not manage the parallelization based on specific options. If a given parameter in the option file specifies a certain number of parallel threads (e.g. *bwa aln “-t” option*), and if the SGE options provided do not require the same number of cores, more than one thread per core will be launched, which could have a major impact on the general efficiency of the job (up to server crash) if too much threads per core are requested.

### Comparison with already available pipeline tools

Different pipelines or pipeline development tools exist for NGS data. The graphical systems (such as *Galaxy* [12], *Taverna* [13], *Tavaxy* [14]) are limited in their options or the available tools, and are generally not able to reach the scale of thousands of samples. The command-line based systems are more versatile (see Table 2), but harder to use.

**Table 2:** Comparison with the different pipelining systems available

Function	TOGGLE	*GATK-Queue*	*HugeSeq*	*Churchill*	*GotCloud*	*bcbio-nextgen*
Raw FASTQ Data	Y	N	N	N	N	Y^a^
Reference indexing	Y	N	N	N	N	Y^a^
FASTQ to final Data	Y	N	Y	Y	Y	Y
Multiple Samples Calling	Y	Y	Y	Y	Y	Y
Modularity	Y	Y	N	N	Y	Y
Between Sample Paralellization	Y	Y	Y	Y	Y	Y
Within Sample Parallelization	Y/N^b^	Y^b^	Y	Y	Y	Y
Structural Variant Analysis	N	N	N	Y	Y	N
Annotation	Y^a^	Y	N	Y	Y	Y
Scalability	Y	Y	N	Y	Y	Y
Evolutivity	Y	Y	Y	Y	Y	Y

*Y* signals that a feature is present, *N* is absent. *Evolutivity* means the possibility to add future module or functions to the system

^a^Available as separated steps

^b^Depending the considered step

*GATK-Queue* [6] is a scripting framework for the *Genome Analysis ToolKit*, which treats *SAM/BAM* or *VCF* files using *GATK* tools. *GATK-Queue* is based on the *Scala* language, and can create simple scripts. However, it is limited to *GATK* tools and will start only after mapping.

The *HugeSeq* pipeline [4] uses a *MapReduce*-like approach on HPC clusters to optimize the calculation of genomic variants from a set of a few already cleaned *FASTQ* data. The scripts are fixed, and parameters can be changed only in hard code modifications or through an extensive and complex command-line. Moreover, a lot of external commands have to be performed before analyses (e.g. creation of reference index and dictionary).

*Churchill* [3] also uses an embarassingly parallel approach in order to achieve *MapReduce* performance. It is dedicated mainly to identification of genomic variants, and scripts are hard coded and can not be modified. It is possible to switch between different mappers and variant callers using a dedicated option file. However, the index and dictionaries for the references have to be created independently, and pair recognition is based only on file names. The whole path for all *FASTQ* files must be provided, and not all options are available for all softwares. Finally, *Churchill* is freely available for academic purposes, as the other ones, but is not open-source.

*GotCloud* [15] is a set of pipelines dedicated to massive NGS analyses, from already cleaned sequence data to association results. It is dedicated to detection of genomics variations, and can switch between mappers or callers. The options system is also based on a single file, but requires that the user knows the *GotCloud* version for each software option. Moreover, it runs only on clusters or in the Cloud (an Amazon EC2 instance is possible), and the name of all *FASTQ* files has to be provided.

The *bcbio-nextgen* framework [16], written in *Python*, is highly modular, and is similar to TOGGLE. Different mappers and callers are already available, and others can be add easily. The framework can be used for germline variants, somatic variants and RNA-Seq, as for TOGGLE. However, the reference sequences are limitated to a fixed set, and even if it is possible to add new items, the system requests those references to be listed.

Compared to the similar frameworks currently available, the current implementations of TOGGLE scripts work on DNA and RNA, and begin with a set of raw *FASTQ* in a given folder. It will automate all steps, from identification of pairs (if any), creation of indexes and dictionaries for the reference, data cleaning and so on. The end-users only have to modify the basic option file *software.config.txt*, in which the very same options as for the command line softwares need to be written. TOGGLE can be extended easily for new bricks and software plugins, and new scripts for dedicated analyses can be assembled quickly. Moreover, TOGGLE can be installed in the user space (without admin rights) as well as at the system level, from a simple laptop to a HPC cluster or in the Cloud.

### Installation

Power users can install TOGGLE following the manual provided on GitHub https://github.com/SouthGreenPlatform/TOGGLE. We are aware that installing all dependencies and working versions of a such large number of softwares is generally problematic and complex. Therefore, we provide alternative methods for installation, a *Docker* image (available at http://bioinfo-web.mpl.ird.fr/toggle/toggle.tgz) and a *bash* script (available on the GitHub and at http://bioinfo-web.mpl.ird.fr/toggle/TOGGLEinstall.sh). The *Docker* image, based on the basic Ubuntu 14.04 image from *Docker* Hub, is ready to run *Out of the box* any NGS data transferred in the container. The *bash* script can be run at user space level and will download all the versions that work with the current version of TOGGLE, and will adapt the different paths for TOGGLE to work. Nevertheless, for an optimized and stable installation, we recommend a more precise installation as detailed on GitHub.

## Conclusion

TOGGLE is a combination of tools for large-scale NGS analyses of DNA and RNAseq data. Users can easily modify the different options of the numerous softwares using the *software.config.txt* file, without going through the code for each step. Users who want to use the general scripts provided in the suite just have to launch the *globalAnalysis.pl* pipeline on the raw *FASTQ* files (in a single folder) and wait to get the *BAM* and *VCF* files. The efficient log files provide information and statistics on the data and pipeline progress quickly, as well as on potential errors or warnings. Thus, users can concentrate on the biological relevance of the results rather than on technical aspects, even if using a huge amount of data.

Bioinformaticians can rapidly write new packages and modules useful for Biologists using a highly reliable system. The addition of new modules is fast, and they can be easily linked to the logging system. A lot of tools are available already, and we will add new modules for structural variant analyses, with softwares such as *NovelSeq* [17] or *BreakDancer* [18] in the near future.

## Availability and requirement

- **Project name:** TOGGLE
- **Project home page:**https://github.com/SouthGreenPlatform/TOGGLE
- **Operating system:** Linux
- **Programming Language:***Perl*
- **Other requirements:***Java* 1.7, *bwa* 0.7.2 or higher, *SAMtools* 0.1.18 or higher, *CutAdapt* 1.2.1 or higher, *FastqC* 0.11.1 or higher, *PicardTools* 1.124 or higher, GATK 3.3x or higher, *Perl* modules: *Data::Dumper*, *Data::Translate*, *Test:More*, *Test::Deep*, *Capture::Tiny*.
- **License:** GNU GPLv3/CeCill-C

## Additional file

Additional file 1
**Complete scheme of the** ***globalAnalysis.pl*** **script.** Colored boxes correspond to analysis steps, with color related to a given package (see text for more information). White and black boxes correspond to input/output files. The *globalAnalysis.pl* script will determine the state of each sample (single or pair), then launch the corresponding subscript, and finally will gather all corrected BAM files to the *mergeAnalysis.pl* script that will perform the multiple sample calling. (PDF 60 kb)
