# Supplementary figures and images for: TOGGLE: toolbox for generic NGS analyses
Source: BMC Bioinformatics. 2015 Nov 9;16:374. doi: 10.1186/s12859-015-0795-6 (PMC4640241; doi:10.1186/s12859-015-0795-6)

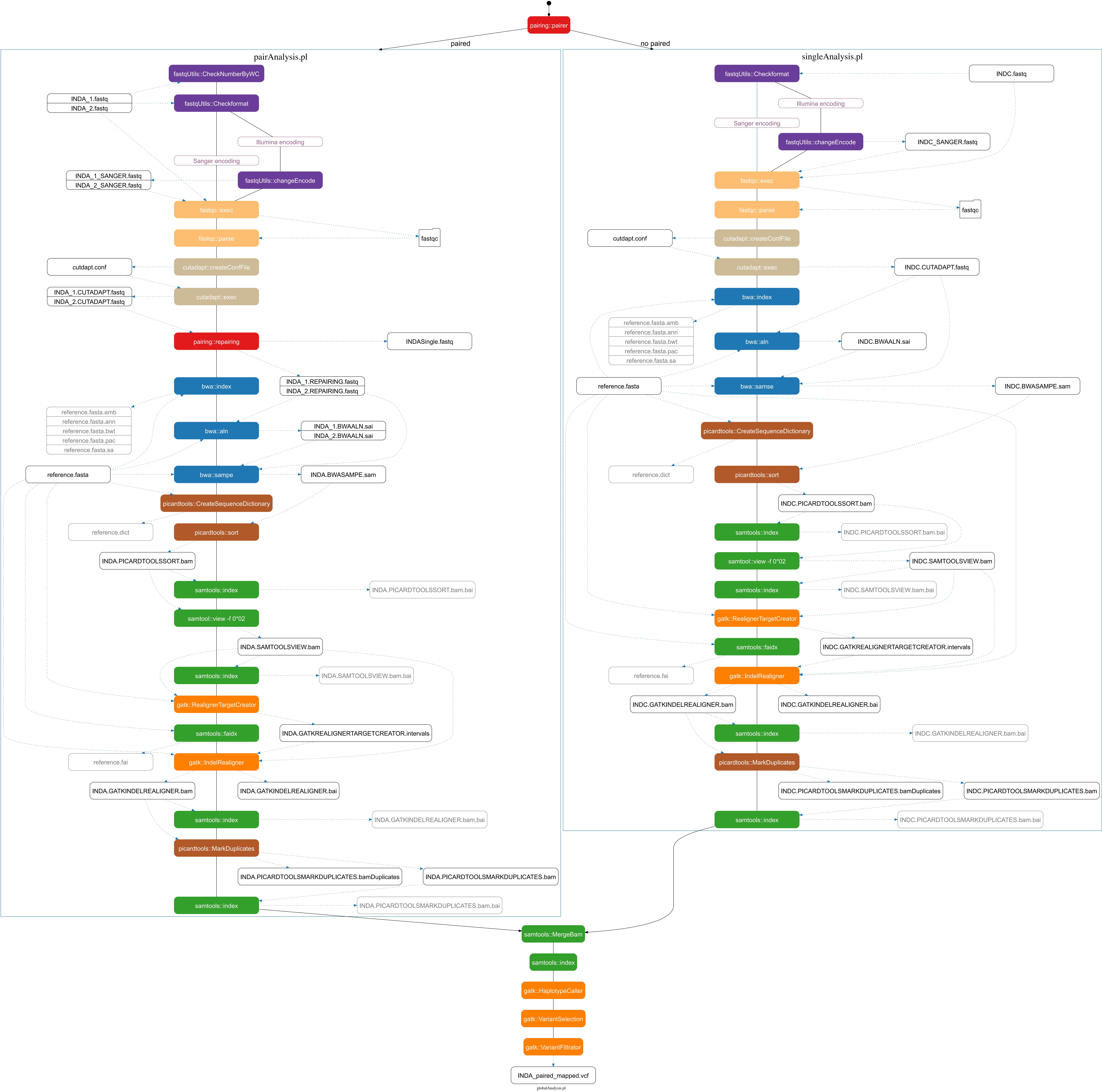

Supplement: Additional file 1 — Complete scheme of the globalAnalysis.pl script. Colored boxes correspond to analysis steps, with color related to a given package (see text for more information). White and black boxes correspond to input/output files. The globalAnalysis.pl script will determine the state of each sample (single or pair), then launch the corresponding subscript, and finally will gather all corrected BAM files to the mergeAnalysis.pl script that will perform the multiple sample calling. (PDF 60 kb) [file 12859_2015_795_MOESM1_ESM.pdf]
